# Consecutive Case Series of Healed Single-Molar Sites Immediately Restored with Wide-Diameter Implants: A 1-Year Evaluation

**DOI:** 10.1155/2016/5645892

**Published:** 2016-04-18

**Authors:** Hadi Antoun, Pierre Cherfane, Bouchra Sojod

**Affiliations:** ^1^Training Institute for Advanced Implant Surgery (IFCIA), 75017 Paris, France; ^2^Department of Oral Surgery and Oral Implantology, School of Dentistry, University of Paris 7, 75013 Paris, France; ^3^Groupe Hospitalier Pitie-Salpetriere, 47-83 Boulevard de l'Hôpital, 75013 Paris, France

## Abstract

*Introduction*. To evaluate outcomes of wide-diameter (6 mm) implants immediately provisionalized with cement-retained single crowns in posterior molar sites.* Materials and Methods*. Forty-eight consecutive patients received a total of 53 moderately rough-surface, 6 mm diameter implants in healed sites. All implants were immediately provisionalized with a cement-retained provisional crown. Final prosthesis with cement-retained porcelain fused to metal crowns was delivered 3–6 months later. Patients were followed up for 1 year. Outcome measures were implant failures and success rate, complications, marginal bone levels, bone level changes, papilla index, bleeding on probing, and inflammation.* Results*. One patient was lost to follow-up. At one year, the implant survival and success rate were 98.1%. The mean marginal bone loss after 1 year was −0.17 ± 1.84 mm. Ideal papilla score was recorded at 83.8% of the sites. More than 95.6% of the sites showed no bleeding or inflammation. No procedure-related or device-related adverse events were reported.* Conclusion*. Wide-diameter (6 mm) implants can safely and successfully replace single posterior molars. Longer follow-up studies are necessary to evaluate the long-term success of these implants.

## 1. Introduction

Implant placement in the posterior region of the jaw presents great occlusal and surgical challenges [1–4]. For a successful single-molar replacement, a balance between functional and parafunctional forces and the capacity of the implant to support prosthesis is critical; otherwise, increased load conditions can develop [5]. The second molars carry the maximum masticatory load, which decreases progressively towards the anterior region of the jaw [1]. For natural teeth, periodontal ligaments help balance the load, but in the case of an implant, the lack of that built-in support can lead to technical complications such as screw or implant fracture [6].

Wide-diameter (WD) implants tolerate higher occlusal forces [7] and offer greater surface area for osseointegration compared with other types of implants, allowing them to provide a high degree of stability and controlled loading conditions even in immediate loading protocols. Indeed, the few studies that investigated bone level changes around implants of a diameter of 6 mm or wider show that no implants had a dramatic bone loss extending past the first implant thread [8–10] or report a remodeling range of −0.24 mm to −0.04 mm [11–13].

Formerly, an alternative way to provide sufficient support for high occlusal forces was to replace a single molar with two implants to mimic the tooth's natural anatomy [14, 15]. However, that option was very difficult in regions with low bone density, limited accessibility for surgical and prosthetic procedures, or insufficient space between adjacent teeth [16]. Additionally, that option limited cleaning access. Therefore, the application of WD implants in smaller molar spaces (8–11 mm) with a crestal width ≥8 mm is of particular interest [17]. Indeed, Degidi et al. reported that WD implants created a wider base for proper prosthesis, were a successful alternative to using two regular-diameter implants for restoration, and were beneficial in the long-term maintenance of various implant-supported prostheses [18]. Surprisingly, there is a scarcity of publications reporting clinical outcomes of implants with a diameter of 6 mm or wider, with only 5 studies replacing single mandibular or maxillary molars [19].

Another factor to consider when restoring first and second molars is the time of loading. Immediate implant loading in such situations has attracted increasing interest among clinicians [16, 20–23]. WD implants can offer high initial stability [24] and therefore might be an effective therapeutic choice in immediate loading protocols. However, there have been few well-designed clinical trials comparing different placement and loading conditions in the posterior regions of the jaw [25]. In that setting, the outcomes of WD implants are conflicting. Schincaglia et al. showed that, compared with delayed loading, immediate loading of 5 mm wide implants resulted in significantly less crestal bone loss after 12 months [4]. Conversely, the only study that included immediately loaded implants of a diameter wider than 5 mm demonstrated no significant clinical or radiographic differences between immediate and delayed loading during a 5-year follow-up [13].

The aim of this clinical study is to report the survival and success of as well as tissue responses to 6 mm implants placed in the mandible or maxilla with immediate provisionalization.

## 2. Materials and Methods

### 2.1. Patients and Study Protocol

This was a single-center case series evaluating the use of 6 mm diameter implants for the replacement of single molars with immediate provisionalization, defined as loading with no static or dynamic occlusion within 48 h of placement. Forty-eight (20 female and 28 male) consecutive patients satisfying the inclusion criteria were prospectively included between April 2007 and October 2010. All participants provided written informed consent to inclusion in the study. The inclusion criteria were as follows: (1) age ≥18 years, (2) fulfilling the general requirements for surgery, (3) missing a single molar or a partially edentulous posterior maxilla or mandible, (4) implantation site with bone height of at least 6 mm beneath the sinus and at least 8 mm above the mandibular canal as diagnosed by panoramic radiograph or CT scan, a posterior jaw crest width of ≥7 mm, and (5) healed implant sites (a minimum of 3-month healing in the maxilla or 4-month healing in the mandible after extraction).

All the patients underwent a complete preoperative evaluation comprising a detailed medical history and clinical and radiographic examinations. Oral hygiene was defined as poor, fair, or good. The participants were classified as smokers or nonsmokers, with no measure of the number of cigarettes consumed per day. A clinical examination of each patient was carried out, and the following information was recorded: date of extraction, mesiodistal distance, prosthetic distance, amplitude of mouth opening, dental and periodontal status, and type of occlusion. A radiographic examination including a panoramic radiograph and cone-beam computed tomography imaging was carried out to assess the volume of the residual bone. Prior to surgery, all the patients underwent conventional periodontal treatment. Initial periodontal therapy was administered to 14 patients who were diagnosed with chronic periodontitis.

### 2.2. Pharmacological Treatment Associated with the Surgical Procedure

A dose of Arnica 9CH (Boiron, Messimy, France) was given the night before and on the first 2 days following surgery. Oral bromazepam (3 mg, Lexomil®, Roche, Boulogne Billancourt, France) was given for sedation 1 h before surgery, and the patients were instructed to rinse with 0.12% chlorhexidine digluconate (Paroex®, Sunstar, Levallois-Perret, France) mouthwash for 1 min immediately before surgery. The patients were instructed to continue with chlorhexidine rinses thrice daily for 2 weeks after the surgery. Implant surgery was performed using local anesthesia with 4% articaine hydrochloride and epinephrine 1/100000 injection (Septanest, Septodont, Saint-Maur des Fosses, France). For 6 days after the surgery, the patients were given 2 g amoxicillin once daily for prophylaxis (Amoxicilline Biogaran®, GlaxoSmithKline, Marly-le-roi, France). Patients who were allergic to penicillin were given 600 mg clindamycin (Dalacin®C 600 mg, Pfizer, Paris, France).

### 2.3. Surgical Protocol

Implants with a 6 mm diameter and varying lengths (range, 7–13 mm; NobelSpeedy Groovy WP, Nobel Biocare, Göteborg, Sweden) were placed at the height of the surrounding bone according to the manufacturer's instructions. The implants were placed with an insertion torque between 20 Ncm and 50 Ncm using the immediate loading protocol. The insertion torque was <30 Ncm in three patients; however, the edentate area in those cases was intercalated by teeth and the temporary crown was out of static and dynamic occlusion. Hence, the risk of jeopardizing osseointegration was deemed very low. Immediately after implant placement, Snappy Abutments WP (1 mm × 4 mm; Nobel Biocare) were connected and tightened up to 35 Ncm.

Flap and flapless implant surgeries were performed. A flap approach was used when the buccal plate was not preserved and the gingival architecture was not harmonious. In those cases, the flap was closed using absorbable sutures (Vicryl 3/0, Johnson & Johnson International, Brussels, Belgium). A flapless approach was used when the buccal plate was preserved and the gingival contour was maintained. Healing caps (Nobel Biocare) were snapped over the abutments. The implant length, bone quality (data not presented), insertion torque, flap design, occurrence of complications, and feasibility of immediate provisionalization were documented at the end of surgery. One week after surgery, all the surgical sites were examined and the sutures were removed in patients who had undergone flap surgery.

### 2.4. Prosthetic Protocol

#### 2.4.1. Temporary Crown

An example of a provisional crown placement is shown in Figure 2. All provisionals were cement-retained acrylic crowns delivered within 48 hours of implant placement. Crowns were constructed over the abutment analog using photopolymerized, injectable composite (Restautomix, Elsodont, Cergy-Pontoise, France). Medical-grade Vaseline (Vaseline Officinale Cooper®, Melun, France) was applied over the sutures and a temporary, light-cured composite Protemp crown (3M ESPE, Cergy-Pontoise, France) was adjusted in the mouth. After the healing cap was removed, the temporary crown was adjusted and tightly adapted on the occlusal, cervical, and proximal portions. Curing light was applied for 3 s after the crown margins were adjusted. The provisional crown was then removed, cleaned, and dried. The prefabricated cap was placed on the abutment to prevent interference with the flap. A small amount of acrylic resin was added to the provisional crown, which was then placed over the cap. Occlusion was checked with a thick articulating paper (200 *μ*m), and all static and dynamic occlusal contacts were eliminated. After complete polymerization, the crown and the cap formed a single entity, which was removed and placed on the abutment. Acrylic resin was used to achieve properly sealed margins between the cap and the Protemp crown, and a proper emergence profile (concave and not compressive) was established. The provisional crown was meticulously polished with acrylic burs and perforated occlusally in the maxilla and lingually in the mandible to evacuate excess cement. A thin layer of polycarboxylate cement (Durelon, 3M ESPE) was applied to the temporary crown, which was then seated on the abutment. All visible excess cement was removed with a dental probe. All patients were advised to follow a soft diet and avoid chewing on the temporary crown for 6 weeks.

#### 2.4.2. Definitive Crown

The final prosthesis was delivered 3–6 months after implantation surgery. All final restorations were composed of cement-retained porcelain fused to metal crowns. The final prosthesis was placed immediately onto the Snappy abutment inserted after the surgery. Prior to cementing, the screw access hole of the Snappy abutment was occluded with a cotton pellet and a temporary light curing material (Fermit, 3M Espe). A retraction cord (Ultrapak®, Ultradent Products, Köln-Porz, Germany) was then packed in the sulcus around the abutment, and a layer of Vaseline was applied around the cervical third of the definitive ceramic crown. A thin layer of polycarboxylate cement was applied to the crown away from the cervical margins. The crown was then seated onto the abutment with finger pressure. Excess cement was eliminated prior to complete setting, and the retraction cord was removed. Further clean-up of the cement margins was accomplished with an explorer #23 (Hu-Friedy, Chicago, IL, USA) and dental floss. The occlusion was checked, and complete seating of the crown was confirmed with an individualized X-ray using a previously prepared silicone bite register.

### 2.5. Measured Variables

A surviving implant was defined as an implant that remained in the jaw and was in function throughout the follow-up period. The implant success rate was evaluated according to van Steenberghe criteria [26], which define a “successful implant” as one that (a) does not cause allergic, toxic, or gross infectious reactions; (b) anchors to a functional prosthesis; (c) shows no signs of fracture or bending; (d) shows no signs of peri-implant radiolucency; (e) is not mobile when tapped or rocked. At the time of surgery (baseline) and at each follow-up visit, the patients were assessed radiographically for bone levels and clinically for occlusion, bleeding on probing, and papilla volume (papilla index according to Jemt, where 0 = no papilla present, 1 = less than half of the papilla height, 2 = half or more of the papilla height, 3 = optimal soft tissue contour with papilla filling up the entire proximal space, and 4 = hyperplastic papilla covering too much of the restoration and/or adjacent tooth) [27]. Visual signs of inflammation were also assessed at baseline and at the follow-up visits. All measurements were performed at follow-up visits 3, 6, and 12 months after implant insertion.

### 2.6. Radiographic Assessments

Periapical radiographs were taken using the long cone parallel technique with a positioner parallel to the long axis of the implant and perpendicular to the X-ray central cone. A silicone bite record with a print of the occlusal side of the teeth was placed on the positioner, which enabled the control X-ray to be repositioned at the follow-up visits. The marginal bone level was evaluated on the basis of the periapical radiographs, only if the implant platform and threads were clearly visible and perpendicular to the X-ray cone. An independent radiologist (University of Leuven) made the measurements. The change in mesial and distal marginal bone levels over time was assessed by comparing the intraoral radiographs taken at implant insertion with those taken at the follow-up visits. The marginal bone level was assessed at the mesial and distal sides of each implant. A reference level was marked at the implant platform, and the bone level was subsequently measured from the reference level to the first point of bone-to-implant contact using Adobe Photoshop software (Adobe System Incorporated, San Jose, CA, USA). The measurements were initially made in a pixel format. Linear measurements (mm) were performed after the images were calibrated according to the respective implant lengths. The bone level was also measured at the distal aspect of the neighboring tooth, if the neighboring tooth was visualized on the same radiograph. The first bone-to-implant contact evaluated by the radiograph taken at implant insertion was defined as the baseline. Marginal bone remodeling was calculated as the difference between the baseline and the readings taken at the follow-up examinations. The measurements of mesial and distal bone height were averaged for each implant. All calculations of the bone levels and of the changes in bone levels were performed using the SAS system version 9 (SAS Institute, Cary, NC, USA).

### 2.7. Clinical Assessments

Bone quality was assessed during surgery according to the criteria described by Lekholm and Zarb [28]. Papillae were assessed using Jemt's papilla index [27]. Bleeding on superficial probing was noted. Signs of inflammation were recorded after visual inspection.

### 2.8. Methodological Aspects

To calculate the cumulative survival rate (CSR) at the patient level, a patient was considered to have experienced failure if at least one of his or her implants failed. The implant-level CSR and all other outcomes were analyzed using the implant as the statistical unit.

## 3. Results

At the time of surgery, the mean patient age was 54 years (range 24 to 79), with ten patients younger than 40 years, thirty-one patients aged between 41 and 70 years, six between 71 and 78 years, and one older than 79 years. The majority (81%) of the patients were nonsmokers. Oral hygiene at the time of inclusion in the study was judged as poor, fair, and good in 31%, 44%, and 25% of the patients, respectively. The patients were followed up clinically and radiologically for 1 year. One patient dropped out after 3 months due to relocation to another country.

Nineteen implants were placed in the maxilla (12 in the first-molar area and 7 in the second-molar area) and 34 were placed in the mandible (27 in the first-molar area and 7 in the second-molar area). Forty-four patients received a single implant, while three received two implants, and one received three implants. Two patients received two implants in neighboring areas (sites 2 and 3), one received two implants in different bones (sites 14 and 19), and one received three implants, two in neighboring areas (sites 16 and 17) and one in another bone (site 30). All the implants had a diameter of 6 mm and a length varying between 7 mm and 13 mm (Table 1). Twenty-one patients underwent flapless surgery, and the remaining patients underwent flap surgery. The bone quality was type II or III for 90.6% of the implants, type I for 5.7% of the implants, and type IV for 3.8% of the implants. The mean insertion torque was 39 ± 6.7 Ncm (Table 1). All the implants were immediately provisionalized (within 48 h of placement).

### 3.1. Implant Survival and Success

The overall implant-level survival rate after 1 year was 98.1% (97.9% at the patient level; Table 2). One implant failed in a 31-year-old, nonsmoking male 44 days after placement in the maxillary first-molar area. The failed implant was short (8.5 mm), had an insertion torque of 25 Ncm, and failed to integrate. The patient received a 6 mm wide replacement implant, which was not immediately provisionalized, and did not experience further complications. All surviving implants were judged as successful at 1-year follow-up, yielding the implant success rate of 98.1%.

### 3.2. Radiographic Outcomes

The mean marginal bone level was −0.56 ± 1.60 mm at baseline and dropped to −0.92 ± 0.81 mm after 3 months and to −1.06 ± 0.72 mm after 6 months, after which it stabilized showing a value of −0.92 ± 0.54 mm at 1 year (Table 3). The marginal bone remodeling from implant insertion to 3, 6, and 12 months after surgery was −0.20 ± 1.57 mm, −0.45 ± 0.66 mm, and −0.17 ± 1.84 mm, respectively. The degree of bone remodeling among the patients at the different follow-up times is shown in Table 4.

### 3.3. Clinical Outcomes

The papilla index score was three (considered ideal) for 83.8% of the implant sites and two for the remaining sites. More than 95.6% of the sites showed no bleeding on probing and no visual signs of inflammation after 1 year.

**Figure 1:**
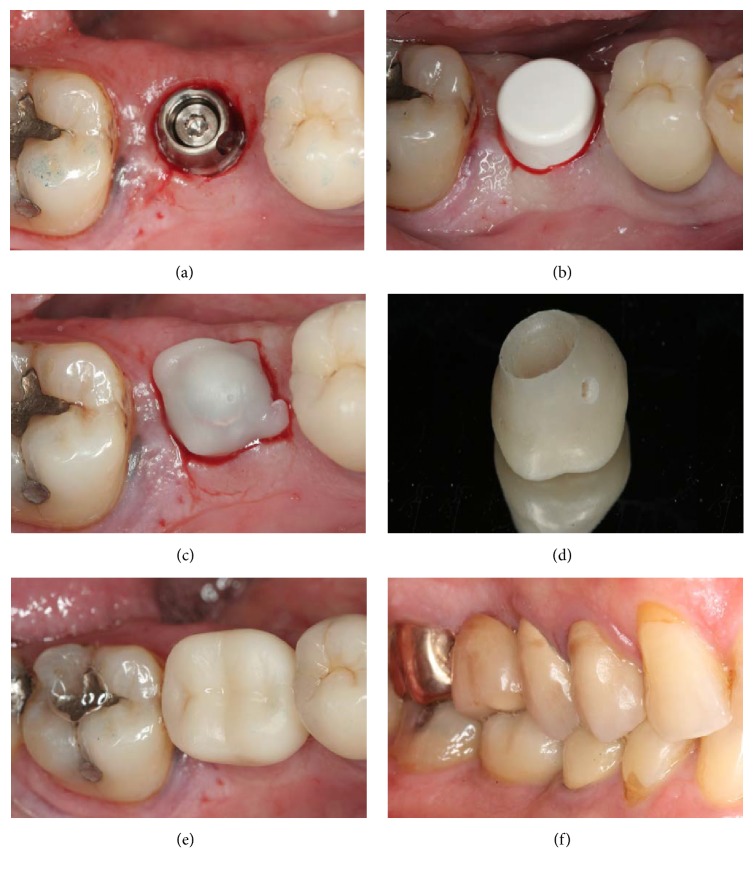
Immediate provisionalization. Occlusal view of the Snappy abutment (a) and the healing cap (b) directly after implant placement using the flapless technique. (c) A prefabricated cap placed over the abutment. (d) The meticulously polished provisional crown with a visible perforation to evacuate excess cement. (e) Occlusal view of the provisional crown after placement, elimination of excess cement, and subsequent complete polymerization. (f) Vestibular view at final prosthesis insertion.

**Figure 2:**
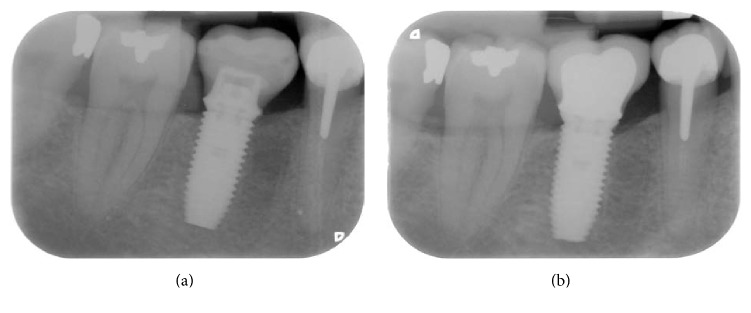
X-ray radiographs of the implant and the restoration (a) at implant insertion and (b) 1 year after surgery.

None of the patients experienced serious or minor procedure-related or device-related adverse events during the 1-year follow-up period. The treatment sequence for one patient is shown in Figures 1 and 2.

## 4. Discussion

This study demonstrated a good overall cumulative implant survival rate 1 year after implant insertion using WD implants in support of immediate restorations in the posterior mandible or maxilla. There were no failures in the mandible after 1 year of follow-up. The single failed implant was located at the first molar in the maxilla and had a low primary stability of 25 Ncm. This study strengthens the previous findings of the few published reports on implants with a diameter of 6 mm or wider, where such implants demonstrated good survival and bone health [19] while contributing new information on soft tissue response to these implants in the context of immediate provisionalization.

This study's findings are in agreement with those of previous studies using moderately rough-surface wide-diameter TiUnite implants. Rao and Benzi reported a 100% CSR after 1 year for 51 implants placed to support single mandibular first-molar screw-retained restorations. Not all of the implants in that study were WD implants; however, 16 had a diameter of 5 mm, and two had a diameter of 6 mm [29]. In another report, Calandriello and Tomatis demonstrated a 95% CSR after 5 years for 5 mm diameter implants placed in immediate function with screw-retained or cement-retained restorations to replace single lower molars [30]. Mura reported a 100% survival rate after 5 years for 47 implants, including 21 with a 5 mm diameter and three with a 6 mm diameter, supporting screw-retained restorations [31]. In a randomized control study of fifteen 5 mm wide WD molar replacements including both cement-retained and screw-retained restorations, Schincaglia et al. reported a 100% CSR for implants with delayed loading and a 93.3% CSR for implants with immediate loading [4].

The overall CSR in our study was higher than those reported in several previous studies of machined-surface WD implants. For example, 5 mm diameter, Brånemark implants had reported CSRs of 76.3% and 80.9% after 5 years [32, 33]; and 5 mm diameter, MKII implants had a reported CSR of 89.8% after 33 months [3]. In another study of 85 WD MKII implants, 19% and 29% of the implants were lost in the mandible and maxilla, respectively [34]. It is unclear, however, whether these differences can be attributed to the differences in implant design, implant surface, or both.

The minimal crestal bone loss in this study was −0.20 mm after 1 year (Table 4), a result considerably better than the results reported in two other studies of moderately rough-surface TiUnite WD implants. Specifically, Schingalia et al. reported −0.77 ± 0.38 mm bone loss 1 year after an immediate loading protocol [4], and Calandriello and Tomatis reported a mean marginal bone loss of −0.73 + 0.43 mm after 1 year [30]. However, among the three studies that were performed on implants with a diameter of >5 mm and which measured the bone response, the bone response tended to be smaller and within a similar range as the one reported in this investigation [11–13].

There were no technical or biological complications in our study. That result contrasts with those of other published studies in which a single implant was used to replace a single molar. Balshi et al. reported a 48% incidence of prosthesis mobility or screw loosening [35], and W. Becker and B. E. Becker reported a 38% incidence of screw loosening [36]. Another study reported a high rate of biological complications associated with cement retention [37]. We attribute the difference between our results and the previous results in part to our use of the technique to limit excess cement originally published by Wadhwani and Pineyro in 2012 [38].

Limitations of our study include the strict inclusion criteria, such as the placement of implants only in healed sites and the absence of bone grafting and bone defects. In addition, the patients were rather heterogeneous, allowing for a wide range of ages, smoking habits, and oral hygiene levels. At the same time, the heterogeneity represents the typical situation in private practice.

## 5. Conclusions

Replacing a single missing molar in the posterior region of the jaw with a WD (6 mm) moderately rough-surface implant supported by a cement-retained single crown is a viable treatment option that leads to low bone remodeling after 1 year. The results of this study confirm the safety and effectiveness of applying the immediate provisionalization protocol for such indications. Future long-term investigations are needed to confirm the encouraging short-term effectiveness of using WD implants in the rehabilitation of missing single molars.

## Figures and Tables

**Table 1:** Implant length and insertion torque.

Implant characteristics	Maxilla	Mandible	Total
Length (mm)			
7	3 (5.7%)	0	3 (5.7%)
8.5	5 (9.4%)	2 (3.8%)	7 (13.2%)
10	6 (11.3%)	7 (13.2%)	13 (24.5%)
11.5	4 (7.5%)	17 (32.1%)	21 (39.6%)
13	1 (1.9%)	8 (15.1%)	9 (17.0%)
Total	19 (35.8%)	34 (64.2%)	53 (100%)
Torque			
Mean ± SD (Ncm)	37.1 ± 8.4	40.1 ± 5.3	39 ± 6.7

**Table 2:** Cumulative survival rate (CSR) of the implants.

Time period	Implants	Failed	Withdrawn	CSR (%)
Placement to 4 weeks	53	0	0	100.0
4 weeks to 6 months	53	1	1^*∗*^	98.1
6 months to 1 year	51	0	0	98.1

^*∗*^1 patient withdrew due to relocation outside the country.

**Table 3:** Bone level at implantation and during follow-up.

Bone level (mm)	Insertion		3 months		6 months		12 months	
Mean	−0.56		−0.92		−1.06		−0.92	
SD	1.60		0.81		0.72		0.54	
Total	47		40		33		33	

	*N*	%	*N*	%	*N*	%	*N*	%

2.1 to 3.0	0	0.0	0	0	0	0.0	0	0.0
1.1 to 2.0	3	6.4	0	0	0	0.0	0	0.0
0.1 to 1.0	1	2.1	0	0	0	0.0	0	0.0
0	18	38.3	4	1	1	3.1	1	2.9
−1 to −0.1	19	40.4	21	17	17	43.8	18	52.9
−2 to −1.1	2	4.3	12	13	13	43.8	13	38.2
−3 to −2.1	2	4.3	1	2	2	6.3	2	5.9
−4 to −3.1	0	0.0	2	0	0	3.1	0	0.0
<−4.0	2	4.3	0	0	0	0.0	0	0.0

**Table 4:** Marginal bone remodeling during the study period.

	Insertion to 3 months		Insertion to 6 months		Insertion to 12 months		3 months to 6 months		6 months to 12 months	
Mean (mm)	−0.20		−0.45		−0.17		−0.01		−0.19	
SD (mm)	1.57		0.66		1.84		0.39		0.69	
Total	38		30		34		26		25	

	*N*	%	*N*	%	*N*	%	*N*	%	*N*	%

>3	1	2.6	0	0.0	2	5.9	0	0.0	0	0.0
2.1 to 3.0	1	2.6	0	0.0	0	0.0	0	0.0	1	4.0
1.1 to 2.0	0	0	1	3.3	2	5.9	1	3.8	0	0.0
0.1 to 1.0	3	7.9	1	3.3	3	8.8	8	30.8	8	32.0
0	5	13.2	4	13.3	1	2.9	4	15.4	1	4.0
−1 to −0.1	25	65.8	21	70.0	19	55.9	13	50.0	13	52.0
−2 to −1.1	2	5.3	2	6.7	7	20.6	0	0.0	2	8.0
−3 to −2.1	0	0.0	1	3.3	0	0.0	0	0.0	0	0.0
−4 to −3.1	1	2.6	0	0.0	0	0.0	0	0.0	0	0.0
